# Predictors  of  Clinical  Benefit  with  Intra-articular Hyaluronic Acid in Patients with Knee Osteoarthritis - A Narrative Review

**DOI:** 10.2174/0115733971274662240108074038

**Published:** 2024-01-18

**Authors:** Xavier Chevalier, Brendan Sheehan

**Affiliations:** 1 Department of Rheumatology, Hôpital Henri-Mondor, Université Paris X11, Créteil, France;; 2 Department of Orthopaedic Surgery, Dalhousie University (Halifax), Saint John, NB, Canada

**Keywords:** Osteoarthritis, intra-articular hyaluronic acid, viscosupplementation, body mass index, disease severity, intra-articular hyaluronic acid injections

## Abstract

**Background:**

There is conflicting evidence regarding the efficacy of viscosupplementation with intra-articular hyaluronic acid injections in knee osteoarthritis. One possible explanation for the inconsistent findings on its efficacy is that only certain subpopulations of patients benefit from this therapy.

**Objective:**

The purpose of this narrative review is to succinctly summarize the existing data on the predictive factors of clinical response to intra-articular hyaluronic acid to identify the patient profile most likely to benefit from this therapy.

**Methods:**

For this narrative review, a PubMed search was conducted in January 2023, with no date limits, to identify publications reporting predictive factors of response to viscosupplementation using the following terms: hyaluronic acid OR viscosupplem* AND osteoarthritis AND knee AND predict*. Searches were limited to randomized controlled trials, systematic reviews and meta-analyses, or observational studies written in English. Other relevant references were identified by searching the references of retrieved articles.

**Results:**

The disease severity was found to reliably predict response to intra-articular hyaluronic acid injections; patients with less severe disease consistently had a more robust therapeutic response than those with more severe disease. Other clinical variables such as level of baseline pain did not reliably predict response. Body mass index, and possibly age, may also be independent predictors of the response.

**Conclusion:**

A review of the existing literature suggests that patients with less severe clinical symptoms and radiological findings, who are younger, and with a lower or normal body mass index are the best candidates for intra-articular hyaluronic acid therapy.

## 1 BACKGROUND

Over the next decade, the prevalence of knee osteoarthritis (KOA) is expected to rise owing to the aging population and ongoing obesity epidemic, resulting in a substantial increase in the total economic burden of this condition [1]. As KOA is incurable, effective use of existing treatments is crucial to reduce symptoms of pain, improve or maintain joint function, slow disease progression, and reduce or delay the need for expensive total knee arthroplasty surgeries.

There is conflicting evidence regarding the efficacy of intra-articular hyaluronic acid (IAHA) in KOA and discrepancies between various clinical practice guidelines on its use [2]. While several international associations endorse IAHA injections [3-7], others state that there is insufficient evidence to support such a recommendation [8-11] One possible explanation underlying the inconsistent findings on IAHA efficacy is that only certain subpopulations of KOA patients benefit from this therapy.

The goal of this narrative review is to summarize the existing data on which patient subgroups are more likely to respond well to IAHA treatment. To this end, a series of PubMed searches were conducted between September 2020 and January 2023 using terms such as hyaluronic acid (hyaluronic acid OR viscosupplem*), osteoarthritis of the knee (osteoarthritis AND knee), and predictive factors (predict*) to identify publications of interest. Searches were limited to randomized trials, systematic reviews, meta-analyses, and observational studies conducted in humans. No publication date filter was applied to the search. Any publication that reported potential predictive factors of good therapeutic response to (or failure of) IAHA therapy was considered for inclusion. Reported outcomes of interest were changes in the pain visual analogue scale (VAS), Western Ontario and McMaster Universities Osteoarthritis Index (WOMAC) scores, International Knee Documentation Committee (IKDC), and Outcome Measures in Rheumatology- Osteoarthritis Research Society International (OMERACT- OARSI) responder rates. Retrieved articles that did not meet these criteria were excluded from consideration following a review of the title and abstract. Publications cited within relevant articles, as well as additional studies identified by the authors were also included based on the above criteria. Only articles that were written in English were considered.

## 2 PREDICTORS OF CLINICAL BENEFIT

### 2.1 Radiological Disease Severity

Studies that sought to identify predictors of clinical benefit or failure of viscosupplementation have looked primarily at radiological features. The extent of radiological disease severity has consistently been shown to be a reliable predictor of IAHA treatment response, regardless of how severity is assessed.

#### 2.1.1 Knee Osteoarthritis Severity based on Kellgren- Lawrence Classification

In the randomized, double-blind FLEXX trial, 588 patients with Kellgren-Lawrence (K-L) grade II or III KOA received 3 weekly injections of either saline or purified hyaluronic acid synthesized by bacterial fermentation [12]. A subgroup analysis indicated that those with less severe disease were more likely to experience significant improvement in VAS pain scores. At 26-week follow-up, the difference in least-squares mean from baseline was -37 mm for K-L II patients receiving IAHA and -22 mm in the intra-articular (IA)-saline group. The -9.7 mm difference between IAHA and IA-saline (95% CI, -16.0 to -3.4 mm) was statistically significant (*p* = 0.003). In contrast, patients with K-L grade III experienced more modest effects and the -4.6 mm difference between treatment with IAHA and IA-saline and did not reach statistical significance (*p* = 0.100, 95% CI, -10.0 to 0.9 mm). The authors suggest that the severity of KOA may be useful to identify patients who are most likely to respond to IAHA treatment, as demonstrated by the difference between K-L II and K-L III groups in achieving statistically significant improvement [12]. However, the lack of inclusion of patients with K-L IV is an important limitation of this study. A recent cross-sectional study which included K-L IV patients examined the patients’ perception of the duration of effectiveness of a single injection of a mannitol-combined, cross-linked, extended-release HA [13]. This study found that patients with K-L grade I or II reported a longer duration of effectiveness compared to patients with K-L grade III or IV (62.6 ± 36.4 weeks *versus* 48.9 ± 18.6 weeks, *p* = 0.03). Notably, although patients with mild-to-moderate disease reported a significantly longer duration of effectiveness, patients with advanced disease still had an average duration of effectiveness of close to one year [13].

Smaller-sized studies have reported that higher proportions of patients with moderate KOA experience improvements in VAS and WOMAC pain scores, relative to patients with more severe disease following IAHA treatment [14, 15]. One study of 21 patients that also included patients with K-L IV and that had a similar follow-up to the subgroup analysis of the FLEXX trial, reported that 100% of patients with moderate KOA (K-L II or III and a total WOMAC index score < 15, n = 16 knees) reported clinical improvements with IAHA treatment, compared to 66% in patients with severe (K-L III and a total WOMAC index score 16-31, n = 6 knees) or very severe osteoarthritis (K-L IV and a total WOMAC index score 38-42, n = 2 knees) [14]. Clinical improvement was assessed according to the following WOMAC parameters: 1) lack of pain at rest; 2) painless walking distance increase; 3) joint range increase; 4) walking up stairs and 5) walking downstairs. Another study compared the treatment response of IAHA to intra-articular platelet-rich plasma (IA-PRP) at 1-year follow-up according to the K-L grade [15]. In this study, patients with K-L I (n = 29) responded better to IAHA treatment than patients with K-L III (n = 23) or K-L IV (n = 10) with respect to WOMAC pain and bodily pain scores in the 36-Item Short Form Survey (SF-36), but these improvements were not statistically significant (PRP *p* = 0.47, IAHA *p* = 0.60) [15].

A larger prospective, comparative study examining treatment with IA-PRP (n = 50) or IAHA (n = 100) in patients with cartilage degenerative lesions on magnetic resonance imaging but with no radiographic features of KOA (K-L 0), early KOA (K-L I to III), or severe KOA (K-L IV) found that treatment with either IA-PRP or IAHA resulted in statistically significant improvements from baseline in the clinical outcomes examined at 2 and 6-month follow-up [16]. However, the study reported that outcomes significantly differed based on baseline disease severity and type of hyaluronic acid (HA) received. Patients treated with IAHA received either a high molecular weight (HMW) or a low molecular weight (LMW) HA. Among the LMW HA group, worse IKDC subjective scores were observed at 2 months in patients affected by advanced KOA compared to patients with early KOA (mean IKDC score of 50 *versus* 61, *p* = .002) or patients with cartilage degenerative lesions (mean IKDC score of 50 *versus* 69, *p* = .001). In the HMW HA group, higher EuroQol VAS were found at 2 months in patients with cartilage degenerative lesions compared to both early OA (*p* = .003) or advanced KOA (*p* = .05) [16]. One important caveat to these data is the inclusion of patients with K-L III within the “early KOA” group.

A prospective observational study on 128 patients with an average 27-month follow-up reported that patients with K-L I (n = 26, 25%) or K-L II (n = 32, 31%) were at least twice as likely to respond to HA treatment, compared to patients with KL grade III (n = 44, 43%) [17]. This study assessed pain and function scores using the VAS and WOMAC/Knee Injury and Osteoarthritis Outcome Score (KOOS) surveys. Response to treatment was defined according to the Osteoarthritis Research Society International (OARSI) 2004 criteria. Of the 102 patients who completed the study, 58 (57%) met the criteria for successful response to treatment. Notably, patients with grade K-L I or K-L II were 2.2 times more likely to respond to the injections than those with K-L III (*p* = 0.001) [17].

A meta-analysis of 20 placebo-controlled randomized trials (N range = 12 to 588 patients) reported significant pain relief with IAHA in trials that included patients with early to moderate KOA (K-L I-III) but not in trials that included end-stage disease (K-L I-IV) [18]. For this meta-analysis, studies were stratified into two groups: those that included patients with K-L grade ≤3 were labelled as the “early-moderate KOA” subgroup, whereas studies that also included patients with K-L grade IV (*i.e.* K-L I-IV, with at least 5% of patients identified as having K-L IV) were labelled as the “late KOA” subgroup. In the early-moderate KOA subgroup, the mean change in pain scores from baseline to 4-13-week follow-up (standardized mean difference (SMD) = - 0.30 (95% CI, - 0.44 to - 0.15), *p* < 0.0001) and to 22-27-week follow-up (SMD = - 0.27 (95%, CI - 0.39 to - 0.16), *p* < 0.00001) was statistically significant in favor of IAHA compared to IA-saline [18]. No significant differences were observed in the late KOA subgroup. IAHA was associated with a significantly greater risk of treatment-related adverse events relative to saline in the late KOA subgroup only (relative risk = 1.76, 95% CI 1.16-2.67, *p* = 0.008) [18].

Vincent *et al.* re-analyzed data from a large, multicenter, open-label, real-world study with a 9-month follow-up that examined clinical outcomes in 1177 patients with KOA receiving IAHA [19]. In this new analysis, patients were stratified according to K-L grade. In the intent-to-treat analysis, change in WOMAC index from baseline was statistically significant in all groups (19.8 (K-L I), 19.8(K-L II), 17.8 (K-L III), 14.2 (K-L IV); *p* < 0.001). The OMERACT-OARSI responder rate reached 72%-82% for K-L grades I-III at 6 and 9 months. However, for patients with disease severity of KL IV, the response rate peaked at 47.7% at 6 months [19].

#### 2.1.2 Knee Osteoarthritis Severity based on Ahlback Grade

Studies using the Ahlback grading of osteoarthritis disease severity also reported that patients with mild-to-moderate disease are more likely to experience therapeutic benefits from IAHA [20, 21]. In a retrospective-cohort study of 183 patients (208 knees), the magnitude of the treatment effect with IAHA was greater in patients with Ahlback grade I-II without mechanical problems, than in patients classified as having more severe disease (Ahlback grade III-V) [20]. In the Ahlback grade I-II group, defined by narrowing of the joint space, WOMAC index score improved from 70.46 to 26.55 (*p* < 0.0001), compared to an improvement of 70.19 to 40.38 (*p* < 0.0001) in the Ahlback grade III-IV group (defined by the presence of bony attrition) and 64.71 to 32.67 (*p* < 0.0001) in the Ahlback grade V group (evidence of lateral subluxation). Given these results, the authors suggest that IAHA may be most effective for patients with Ahlback grade I-II [20].

#### 2.1.3 Knee Osteoarthritis Severity based on Joint Space Narrowing

Studies have also looked at specific radiological parameters as predictors of therapeutic response to IAHA. One prospective study of 60 patients examined the correlation between the efficacy of viscosupplementation with the severity of osteoarthritis on roentgenological examination [22]. The authors reported an association between K-L grade (I-IV) and clinical outcomes, including overall WOMAC index (*p* = 0.014), stiffness (0.046), and pain (*p* = 0.018) at 12-week follow-up, but not with functional outcomes [22]. An observer blinded to the outcome of interested graded the severity of osteoarthritic changes as either minor (*i.e*., changes corresponding to minimal to mild KOA, *e.g*., K-L I-II) or major (*i.e*., changes encompassing moderate to severe KOA, *e.g*., K-L III-IV). Examination of specific radiological parameters indicated that relative to patients with major changes, a higher proportion of patients with minor changes in lateral and medial joint space narrowing reported significant improvement in WOMAC function (lateral, 37.0% (minor changes) *versus* 12.1% (major changes), *p* = 0.033; medial, 40.0% *versus* 15.0%, *p* = 0.050), WOMAC pain (lateral, 51.9% (minor changes) *versus* 15.2% (major changes), *p* = 0.005; medial, 75.0% *versus* 10.0%, *p* < 0.001), WOMAC stiffness (lateral, 51.9% (minor changes) *versus* 24.2% (major changes), *p* = 0.034; medial, 65.0% *versus* 22.5%, *p* = 0.002), and WOMAC index (lateral, 37.0% (minor changes) *versus* 6.1% (major changes), *p* = 0.004; medial, 45.0% *versus* 7.5%, *p* = 0.001) scores. Patients with minor tibial spine changes were also more likely to report improvement in WOMAC pain (*p* = 0.022), WOMAC stiffness (*p* < 0.001), and WOMAC index (*p* = 0.009) scores [22].

#### 2.1.4 Knee Osteoarthritis Severity based on Cartilage Volume Measured by Magnetic Resonance Imaging

A retrospective analysis of data from 310 patients from the Osteoarthritis Initiative database was conducted to identify characteristics associated with a better response to IAHA treatment [23]. Patients with baseline WOMAC pain scores of ≥ 8 (n=103, 112 knees) were classified as responders if they demonstrated 20% or greater improvement in pain or as nonresponders if their pain remained unchanged or worsened within the 6 months following treatment. The authors found a significant difference between responders and nonresponders in cartilage volume of the medial compartment (4434 ± 1455 mm^3^ (responders) *versus* 3675 ± 1434 mm^3^ (nonresponders), *p* = 0.046), and suggested that greater cartilage volume may be a predictor of better IAHA treatment response [23].

#### 2.1.5 Knee Osteoarthritis Severity based on OARSI Grade

An osteoarthritis cartilage histopathology assessment system, based on six grades that reflect the depth of the lesion and four stages reflecting extent of osteoarthritis over the joint surface was developed by the Osteoarthritis Research Society International [24, 25]. This OARSI system is composed of a grading and a staging component, wherein a higher grade indicates more aggressive biologic progression, and a higher stage indicates a wider disease extent.

A post-hoc analysis of 166 patients from the HAV-2012 trial grouped according to the OARSI system, revealed that severe KOA is significantly associated with a failure of IAHA to produce favorable therapeutic outcomes [26, 27]. At 6 months, 113 (68.1%) of patients were OMERACT-OARSI responders, defined as a decrease ≥50% and an absolute change ≥20 points in WOMAC pain or function. Multivariate analysis showed that radiological severity was a significant factor predicting OMERACT-OARSI response following IAHA treatment; greater radiological severity (OARSI grade 3) was associated with failure to respond (*p* = 0.008) [26]. A second post-hoc analysis [28] examined pain improvement rather than response rate. In this analysis, patients were stratified according to body mass index (non-obese or obese), OARSI radiological grade (I-II *versus* III), and OMERACT-OARSI response (yes or no). At 6-month follow-up, WOMAC pain scores were significantly improved in the patients with OARSI I-II *versus* III (4.8 ± 4.3 *versus* 6.4 ± 4.5; *p* = 0.009). Interestingly, when the researchers looked only at the population who met the OMERACT- OARSI response criteria, there was no significant difference between patients with OARSI I-II *versus* III [28]. Taken together, these data suggest that while response rates are lower for patients with more severe radiographic disease [27], patients who do respond with severe disease may benefit as greatly as patients with less severe disease [28].

### 2.2 Predictors on Structural Benefit

Evidence of a definitive structural effect of IAHA has yet to be demonstrated. One randomized double-blind, placebo-controlled trial of 534 patients used joint space narrowing (JSN) as a radiological parameter to investigate structural changes during treatment with IAHA over a 52-week period [29]. No statistically significant differences in structural changes between IAHA and placebo were found. However, the study revealed an interaction between baseline joint space width (JSW) and response to treatment, suggesting that treatment outcomes may depend on the extent of joint space narrowing (*p* = 0.01). A subgroup analysis compared patients with a JSW of more than 4.6 mm with those of 4.6 mm or less. In the subgroup of patients with milder disease at baseline, the change in JSN after 52 weeks was significantly reduced in the IAHA group compared to the placebo group (0.13 ± 1.05 mm *versus* 0.55 ± 1.04 mm, *p* = 0.02). This effect, however, was not observed in patients with more severe disease [29]. Similarly, a study assessing factors predictive of good treatment outcomes as measured by patient-reported satisfaction following treatment with an HMW cross-linked HA, found that limited joint space loss (restricted to a single compartment) was associated with a better response to IAHA therapy [30].

## 3 LEVEL OF BASELINE PAIN

There is evidence to suggest that a higher level of pain at baseline may be predictive of a positive response to IAHA therapies. One prospective study of 4253 patients that assessed the tolerability and short-term effectiveness of IAHA demonstrated that patients with severe baseline pain were more likely to have a favorable response to treatment [31]. Compared to patients with severe baseline pain, patients with mild (odds ratio (OR) 0.11, 95% CI 0.08-0.15) and moderate (OR 0.49, 95% CI 0.38-0.64) were significantly less likely to report a decrease in pain. Similarly, the results from the Osteoarthritis Initiative, a prospective cohort study that followed 310 KOA patients for nearly a decade, identified having high levels of knee pain, defined as a WOMAC pain score ≥8, as a predictive determinant of a better response to IAHA treatment [23]. However, the possibility that these data reflect the “regression to the mean” phenomenon cannot be ruled out. The primary objective of these studies was not to establish efficacy of IAHA, but to establish tolerability and did not include a comparison group [23, 31].

In contrast, other studies have not identified the level of baseline pain as a predictor of IAHA response. One prospective study that evaluated 102 patients with mild-to-moderate KOA (K-L I-II) did not find baseline VAS pain score to be a predictor of response to IAHA treatment [17], nor was baseline pain intensity found to be independently associated with treatment response in a post-hoc multivariate analysis (N = 166) of data from a prospective, multicenter, randomized, non-inferiority trial comparing two HA products in symptomatic KOA [26, 27]. Furthermore, a subgroup analysis of a single-center randomized study comparing the tolerability and efficacy of an IAHA (n = 75 patients) *versus* corticosteroids (CS, n = 75 patients) showed that IAHA provided a more sustained reduction in pain compared to corticosteroids (VAS pain scores at 26 weeks; 2.1 ± 1.2 (IAHA) *versus* 4.0 ± 1.3 (CS), *p* < 0.0001) in patients with lower VAS pain scores at baseline (VAS pain score between 3 and 6 points) [32]. This beneficial effect was not observed for patients who reported higher levels of pain at baseline (VAS pain scores at 26 weeks; 6.0 ± 1.6 (IAHA) *versus* 6.0 ± 1.5 (CS), *p* = 1). A secondary linear regression analysis performed on the data from the FLEXX trial and its extension study to examine predictors of pain outcomes at 6 and 12- month follow-ups further support these findings [12, 33]. A higher baseline VAS pain score was associated with having a higher VAS pain score after the 50-foot walk test at both 6 months (*p* = 0.001) and 12 months (*p* < 0.001), suggesting reduced benefit of IAHA treatment in patients with higher level of baseline pain. Conversely, when data were analyzed in terms of response (*i.e.*, OMERACT-OARSI response analysis) rather than the level of pain, the authors reported that at 12-month follow-up, patients with a higher baseline pain score were more likely to be an OMERACT-OARSI responder [33].

## 4 DEMOGRAPHIC CHARACTERISTICS

### 4.1 Body Mass Index/Obesity

Although not conclusive, the current body of literature does suggest that obesity may have a negative impact on the response to IAHA treatment. Data from multiple studies indicate that patients with a lower or normal body mass index (BMI) demonstrate statistically significant improvements in patient reported outcomes such as WOMAC pain and IKDC subjective VAS at varying follow-up time points following IAHA treatment relative to patients with a higher BMI or who are obese [27, 28, 31, 34]. A post-hoc analysis of a prospective randomized, multicenter non-inferiority trial comparing two IAHA products in 226 patients with symptomatic KOA was conducted to identify the demographic, clinical, or radiological factors associated with nonresponse to IAHA according to OMERACT-OARSI criteria [27]. The authors reported that higher BMI and greater JSN were two independent predictors of nonresponse that can also have an additive negative effect. Importantly, the presence of obesity does not necessarily negate the beneficial effects of IAHA treatment as shown in a subsequent analysis of these data [28]. This second post-hoc analysis was conducted to assess the impact of obesity and radiological stage on the efficacy of IAHA in terms of pain improvement and clinical status at 6-month follow-up, and not the response rate. For this analysis, patients were stratified by BMI (obese *versus* non-obese) and by OARSI radiological grade (I-II *versus* III). Notably, all patient subgroups demonstrated significantly decreased WOMAC pain scores from baseline at 6-month follow-up (all *p* < 0.01), even the obese patients (baseline *versus* 6-month follow-up: 10.4 ± 3.1 *versus* 7.1 ± 4.9) [28]. Although the chance of being a “good responder” to IAHA therapy was lower for obese patients, for those who did respond, the benefit was similar to that of patients with a normal BMI (difference in WOMAC pain score from baseline non-obese *versus* obese: -6.9 ± 2.8 *versus* -6.7 ± 3.4, *p* > 0.05), even among those with OARSI radiological grade III KOA (difference in WOMAC pain scores from baseline -6.7 ± 2.3) [28].

### 4.2 Gender

Gender does not appear to reliably predict response to IAHA; one prospective, observational study of over 4000 patients identified male gender as a possible predictor of short-term IAHA effectiveness; relative to females, males were significantly more likely to report a decrease in pain at 3-weeks follow-up (OR 1.45, 95% CI 1.19-1.77) [31]. A smaller study carried out under conditions of daily practice aimed at identifying possible predictive factors of viscosupplementation duration of effectiveness found that men reported a longer duration of effectiveness compared to women [13]. However, other studies have failed to replicate gender differences in efficacy or duration of effectiveness [17, 19, 27, 35].

### 4.3 Age

Although numerous studies indicate that younger age may be an independent, positive predictor of response to IAHA treatment [23, 35-38], others have reported no meaningful differences in response to IAHA treatment between younger and older patients [15, 19, 39]. Indeed, studies have demonstrated that IAHA treatment can result in significant improvements in pain in patients over 60, especially when the confounding variable of disease severity is controlled (*i.e.*., greater probability that older patients have more advanced KOA and therefore may be less likely to respond) [36, 40, 41].

## CONCLUSION

Our review of the literature shows that disease severity is the main predictive factor of a poorer response to IAHA. Although further research is warranted, the existing data consistently demonstrate that intra-articular therapies such as IAHA are most efficacious when used earlier in the disease course. This finding may have important implications on the burgeoning economic burden of KOA, as earlier use of IAHA in appropriate patients may delay the need for total knee arthroplasty (TKA) and significantly reduce healthcare costs [42-52]. A recent systematic review of the economic impact of IAHA for KOA pain concluded that repeated courses of IAHA could not only delay the need for TKA by 5 years, but it was cost-effective against NSAIDs, IACS, analgesics and conservative treatment [49].

Further research is required to explain why greater disease severity is associated with a lesser clinical response to IAHA. The exact mechanisms of action underlying the effect of IAHA have yet to be fully elucidated, but it is likely to involve chondroprotection, proteoglycan/glycosaminoglycan synthesis, and anti-inflammatory effects *via* binding to chondrocyte CD44 receptors on chondrocytes [53]. In human chondrocytes, binding of HA to CD44 results in the inhibition of interleukins (IL)-1β and IL-6, leading to decreased expression of various matrix metalloproteinases (MMP) [54, 55]. Through inhibiting IL-induced MMP activity, IAHA may help protect against cartilage degradation and exert an anti-inflammatory effect [53, 56]. If IAHA exerts its long-lasting effects through HA-CD44 mediated chondroprotection, it stands to reason that IAHA therapy would be most beneficial in patients with less pre-existing articular degradation. Indeed, as demonstrated by Henrotin *et al, *patients who have lower serum levels of Type II collagen degradation product at baseline are more likely to reach OMERACT-OARSI criteria for high responders [57]. Interestingly, binding to CD44 has been shown to be of greater effect for HMW products which may underlie improvements in pain management with HMW IAHAs relative to LMW IAHA [53, 58].

Despite the existing evidence supporting earlier use of IAHA, many patients who could benefit from this therapy are only receiving it later in the disease course, if at all. In the United States, the current continuum of care includes prescription pain medications followed by IACS then IAHA, and finally, TKA [49]. Many insurers will only cover the cost of IAHA after failure of cheaper IACS treatments. As shown in different meta-analyses, recurrent IACS injections are associated with mild improvements in pain, function and quality, however other injectable therapies, including IAHA, provide greater improvements with a longer duration [59, 60]. Furthermore, there is a growing body of evidence that repeated use of IACS may not be as safe as previously thought as possible accelerated OA progression, subchondral insufficiency fractures, osteonecrosis, and/or rapid joint destruction have been reported with repeated use of IACS [61, 62]. In addition, elevations in blood glucose levels have been observed in KOA patients with diabetes following intra-articular injection of some corticosteroids, raising concerns about safety in this patient population [63-65]. Conversely, evidence exists that multiple courses of IAHA injections are not only safe but are also associated with greater improvements than a single course of treatment [66, 67]. Delaying the use of IAHA until failure with IACS results in its use in more advanced stages (*i.e.*, grade IV), and thus likely reduces the potential maximal effectiveness and contributes to the uncertainty of efficacy [68].

The evidence supporting the predictive power of demographic characteristics such as weight and age on clinical response to IAHA is less robust than it is for disease severity. Notably, level of baseline pain does not reliably predict response to IAHA. Although the data for these factors are less consistent, our review of the existing literature does suggest that these are patient characteristics clinicians should consider when determining whether IAHA therapy is appropriate for a particular patient.

For a significant proportion of clinicians, IAHA remains an important treatment of choice for patients with mild-to- moderate KOA [69]. The fact that physicians continue to use IAHA, even though some clinical practice guidelines do not recommend its use, speaks to clinicians’ perspectives on the effectiveness of IAHA in “real-world” clinical settings, particularly for patients with mild-to-moderate KOA [70]. In 2018, the European Viscosupplementation Consensus Group (EUROVISCO) Expert Working Group published a proposed a set of recommendations for optimizing the clinical results of viscosupplementation in osteoarthritis that were based on evidence and on daily practice clinical experience [71]. There was agreement among the expert members of the working group for treating patients with mild-to-moderate KOA, who were normal weight or moderately overweight, insufficiently managed with first-line therapies, or who have contraindications to oral analgesic medications [71].

While further research on the identification of which patient subgroups are most likely to have a robust therapeutic response to IAHA is warranted, our updated review of the existing evidence supports the EUROVISCO recommendations for the use of IAHA therapy in patients with less severe clinical symptoms and radiological findings and with a lower or normal BMI. We also suggest that patients who are younger may exhibit a better clinical response to IAHA therapy.

## AUTHORS’ CONTRIBUTIONS

Both authors contributed to the conception, writing and revisions of the manuscript. The authors have read and approved the final manuscript.

## LIST OF ABBREVIATIONS

BMI: Body Mass Index
CS: Corticosteroids
HA: Hyaluronic Acid
HMW: High Molecular Weight
IAHA: Intra-Articular Hyaluronic Acid
IA-PRP: Intra-Articular Platelet-Rich Plasma
IA-saline: Intra-Articular Saline
IKDC: International Knee Documentation Committee
IL: Interleukin
JSN: Joint Space Narrowing
JSW: Joint Space Width
K-L: Kellgren-Lawrence
KOA: Knee Osteoarthritis
KOOS: Knee Injury and Osteoarthritis Outcome Score
LMW: low Molecular Weight
MMP: Matrix Metalloproteinases
OARSI: Osteoarthritis Research Society International
OMERACT-OARSI: Outcome Measures in Rheumatology-Osteoarthritis Research Society International
OR: Odds Ratio
SMD: Standardized Mean Difference
VAS: Visual Analogue Scale
WOMAC: Western Ontario and McMaster Universities Osteoarthritis Index

## References

[1] Hootman J.M., Helmick C.G., Barbour K.E., Theis K.A., Boring M.A. (2016). Updated projected prevalence of self-reported doctor-diagnosed arthritis and arthritis-attributable activity limitation among US adults, 2015–2040.. Arthritis Rheumatol..

[2] Rosen J., Avram V., Fierlinger A., Niazi F., Sancheti P., Bedi A. (2016). Clinicians’ perspectives on the use of intra-articular hyaluronic acid as a treatment for knee osteoarthritis: A North American, multidisciplinary survey.. Clin. Med. Insights Arthritis Musculoskelet. Disord..

[3] Bannuru R.R., Osani M.C., Vaysbrot E.E., Arden N.K., Bennell K., Bierma-Zeinstra S.M.A., Kraus V.B., Lohmander L.S., Abbott J.H., Bhandari M., Blanco F.J., Espinosa R., Haugen I.K., Lin J., Mandl L.A., Moilanen E., Nakamura N., Snyder-Mackler L., Trojian T., Underwood M., McAlindon T.E. (2019). OARSI guidelines for the non-surgical management of knee, hip, and polyarticular osteoarthritis.. Osteoarthritis Cartilage.

[4] Trojian T.H., Concoff A.L., Joy S.M., Hatzenbuehler J.R., Saulsberry W.J., Coleman C.I. (2016). AMSSM scientific statement concerning viscosupplementation injections for knee osteoarthritis.. Clin. J. Sport Med..

[5] Rillo O., Riera H., Acosta C., Liendo V., Bolaños J., Monterola L., Nieto E., Arape R., Franco L.M., Vera M., Papasidero S., Espinosa R., Esquivel J.A., Souto R., Rossi C., Molina J.F., Salas J., Ballesteros F., Radrigan F., Guibert M., Reyes G., Chico A., Camacho W., Urioste L., Garcia A., Iraheta I., Gutierrez C.E., Aragón R., Duarte M., Gonzalez M., Castañeda O., Angulo J., Coimbra I., Munoz-Louis R., Saenz R., Vallejo C., Briceño J., Acuña R.P., De León A., Reginato A.M., Möller I., Caballero C.V., Quintero M. (2016). PANLAR consensus recommendations for the management in osteoarthritis of hand, hip, and knee.. J. Clin. Rheumatol..

[6] Bruyère O., Cooper C., Pelletier J.P., Branco J., Luisa Brandi M., Guillemin F., Hochberg M.C., Kanis J.A., Kvien T.K., Martel-Pelletier J., Rizzoli R., Silverman S., Reginster J.Y. (2014). An algorithm recommendation for the management of knee osteoarthritis in Europe and internationally: A report from a task force of the European Society for Clinical and Economic Aspects of Osteoporosis and Osteoarthritis (ESCEO).. Semin. Arthritis Rheum..

[7] Bruyère O., Honvo G., Veronese N., Arden N.K., Branco J., Curtis E.M., Al-Daghri N.M., Herrero-Beaumont G., Martel-Pelletier J., Pelletier J.P., Rannou F., Rizzoli R., Roth R., Uebelhart D., Cooper C., Reginster J.Y. (2019). An updated algorithm recommendation for the management of knee osteoarthritis from the European Society for Clinical and Economic Aspects of Osteoporosis, Osteoarthritis and Musculoskeletal Diseases (ESCEO).. Semin. Arthritis Rheum..

[8] American Academy of Orthopaedic Surgeons Management of osteoarthritis of the knee (Non-Arthroplasty) evidence-based clinical practice Guideline.. https://aaos.org/quality/quality-programs/lower-extremity-programs/osteoarthritis-of-the-knee/.

[9] Kolasinski S.L., Neogi T., Hochberg M.C., Oatis C., Guyatt G., Block J., Callahan L., Copenhaver C., Dodge C., Felson D., Gellar K., Harvey W.F., Hawker G., Herzig E., Kwoh C.K., Nelson A.E., Samuels J., Scanzello C., White D., Wise B., Altman R.D., DiRenzo D., Fontanarosa J., Giradi G., Ishimori M., Misra D., Shah A.A., Shmagel A.K., Thoma L.M., Turgunbaev M., Turner A.S., Reston J. (2020). 2019 American College of rheumatology/arthritis foundation guideline for the management of osteoarthritis of the hand, hip, and knee.. Arthritis Care Res..

[10] NICE Osteoarthritis: care and management.. https://www.nice.org.uk/guidance/cg177/evidence/full-guideline-pdf-191761311.

[11] VA/DoD Clinical Practice Guidelines The non-surgical management of hip & knee osteoarthritis (OA) (2020).. https://www.healthquality.va.gov/guidelines/cd/oa/index.asp.

[12] Altman R.D., Rosen J.E., Bloch D.A., Hatoum H.T., Korner P. (2009). A double-blind, randomized, saline-controlled study of the efficacy and safety of EUFLEXXA for treatment of painful osteoarthritis of the knee, with an open-label safety extension (the FLEXX trial).. Semin. Arthritis Rheum..

[13] Perruchet S., Balblanc J.C., Rapp C. (2022). The Association between radiographic features and the duration of effectiveness of a single injection of extended-release hyaluronic acid (HANOX-M-XL) in patients with knee osteoarthritis: Preliminary results of a prospective trial.. Cartilage.

[14] Polacco A., Zobel B.B., Polacco M., Scarlata S., Gasparro F., Del Vescovo R., Scarciolla L. (2013). The effect of intra-articular hyaluronic acid (Sinovial(R) One) on knee osteoarthritis: a preliminary study.. Eur. J. Inflamm..

[15] Raeissadat S.A., Rayegani S.M., Hassanabadi H., Fathi M., Ghorbani E., Babaee M., Azma K. (2015). Knee osteoarthritis injection choices: Platelet- rich plasma (PRP) *versus* hyaluronic acid (A one-year randomized clinical trial).. Clin. Med. Insights Arthritis Musculoskelet. Disord..

[16] Kon E., Mandelbaum B., Buda R., Filardo G., Delcogliano M., Timoncini A., Fornasari P.M., Giannini S., Marcacci M. (2011). Platelet-rich plasma intra-articular injection *versus* hyaluronic acid viscosupplementation as treatments for cartilage pathology: From early degeneration to osteoarthritis.. Arthroscopy.

[17] Bowman E.N., Hallock J.D., Throckmorton T.W., Azar F.M. (2018). Hyaluronic acid injections for osteoarthritis of the knee: predictors of successful treatment.. Int. Orthop..

[18] Nicholls M., Shaw P., Niazi F., Bhandari M., Bedi A. (2019). The impact of excluding patients with end-stage knee disease in intra-articular hyaluronic acid trials: A systematic review and meta-analysis.. Adv. Ther..

[19] Vincent P., Lucas de Couville T., Thomas T. (2020). Intra-articular hyaluronic acid for knee osteoarthritis: A postmarket, open-label, long-term historical control study with analysis detailed per krellgren-lawrence radiologic osteoarthritis scale grade.. Curr. Ther. Res. Clin. Exp..

[20] Turajane T., Tanavaree A., Labpiboonpong V., Maungsiri S. (2007). Outcomes of intra-articular injection of sodium hyaluronate for the treatment of osteoarthritis of the knee.. J. Med. Assoc. Thai..

[21] Guidolin D. (2018). Intra-articular 500-730 kDa hyaluronan (Hyalgan^®^) therapy in the management of osteoarthritis. Can a specific therapeutic profile be defined?. Eur. Rev. Med. Pharmacol. Sci..

[22] Toh E.M., Prasad P.S., Teanby D. (2002). Correlating the efficacy of knee viscosupplementation with osteoarthritic changes on roentgenological examination.. Knee.

[23] Pelletier J.P., Raynauld J.P., Abram F., Dorais M., Delorme P., Martel-Pelletier J. (2018). Exploring determinants predicting response to intra-articular hyaluronic acid treatment in symptomatic knee osteoarthritis: 9-year follow-up data from the Osteoarthritis Initiative.. Arthritis Res. Ther..

[24] Pritzker K.P.H., Gay S., Jimenez S.A., Ostergaard K., Pelletier J.P., Revell P.A., Salter D., van den Berg W.B. (2006). Osteoarthritis cartilage histopathology: Grading and staging.. Osteoarthritis Cartilage.

[25] Custers R.J.H., Creemers L.B., Verbout A.J., van Rijen M.H.P., Dhert W.J.A., Saris D.B.F. (2007). Reliability, reproducibility and variability of the traditional histologic/histochemical grading system *vs* the new OARSI osteoarthritis cartilage histopathology assessment system.. Osteoarthr. Cartil..

[26] Conrozier T., Eymard F., Afif N., Balblanc J.C., Legré-Boyer V., Chevalier X. (2016). Safety and efficacy of intra-articular injections of a combination of hyaluronic acid and mannitol (HAnOX-M) in patients with symptomatic knee osteoarthritis.. Knee.

[27] Eymard F., Chevalier X., Conrozier T. (2017). Obesity and radiological severity are associated with viscosupplementation failure in patients with knee osteoarthritis.. J. Orthop. Res..

[28] Conrozier T., Eymard F., Chouk M., Chevalier X. (2019). Impact of obesity, structural severity and their combination on the efficacy of viscosupplementation in patients with knee osteoarthritis.. BMC Musculoskelet. Disord..

[29] Jubb R.W., Piva S., Beinat L., Dacre J., Gishen P. (2003). A one-year, randomised, placebo (saline) controlled clinical trial of 500-730 kDa sodium hyaluronate (Hyalgan) on the radiological change in osteoarthritis of the knee.. Int. J. Clin. Pract..

[30] Conrozier T., Mathieu P., Schott A.M., Laurent I., Hajri T., Crozes P., Grand P., Laurent H., Marchand F., Meignan F., Noel E., Rozand Y., Savoye J.F., Vignon E. (2003). Factors predicting long-term efficacy of Hylan GF-20 viscosupplementation in knee osteoarthritis.. Joint Bone Spine.

[31] Kemper F., Gebhardt U., Meng T., Murray C. (2005). Tolerability and short-term effectiveness of hylan G-F 20 in 4253 patients with osteoarthritis of the knee in clinical practice.. Curr. Med. Res. Opin..

[32] Bisicchia S., Bernardi G., Tudisco C. (2016). HYADD 4 *versus* methylprednisolone acetate in symptomatic knee osteoarthritis: A single-centre single blind prospective randomised controlled clinical study with 1-year follow-up.. Clin. Exp. Rheumatol..

[33] Altman R.D., Farrokhyar F., Fierlinger A., Niazi F., Rosen J. (2016). Analysis for prognostic factors from a database for the intra-articular hyaluronic acid (Euflexxa) treatment for osteoarthritis of the knee.. Cartilage.

[34] Cole B.J., Karas V., Hussey K., Merkow D.B., Pilz K., Fortier L.A. (2017). Hyaluronic acid *versus* platelet-rich plasma: A prospective, double-blind randomized controlled trial comparing clinical outcomes and effects on intra-articular biology for the treatment of knee osteoarthritis.. Am. J. Sports Med..

[35] Kearey P., Popple A.E., Warren J., Davis T., Bellamy N. (2017). Improvement in condition-specific and generic quality of life outcomes in patients with knee osteoarthritis following single-injection Synvisc: results from the LOBRAS study.. Curr. Med. Res. Opin..

[36] Wang C.T., Lin J., Chang C.J., Lin Y.T., Hou S.M. (2004). Therapeutic effects of hyaluronic acid on osteoarthritis of the knee. A meta-analysis of randomized controlled trials.. J. Bone Joint Surg. Am..

[37] Uçar D., Dıraçoğlu D., Süleyman T., Çapan N. (2013). Intra-articular hyaluronic Acid as treatment in elderly and middle-aged patients with knee osteoarthritis.. Open Rheumatol. J..

[38] Ong K., Lau E., Runa M., Daley W., Altman R. (2021). Factors associated with knee arthroplasty in a knee osteoarthritis patient cohort treated with intra-articular injections of hylan G-F 20.. J. Knee Surg..

[39] Jørgensen A., Stengaard-Pedersen K., Simonsen O., Pfeiffer-Jensen M., Eriksen C., Bliddal H., Pedersen N.W., Bødtker S., Hørslev-Petersen K., Snerum L.Ø., Egund N., Frimer-Larsen H. (2010). Intra-articular hyaluronan is without clinical effect in knee osteoarthritis: a multicentre, randomised, placebo-controlled, double-blind study of 337 patients followed for 1 year.. Ann. Rheum. Dis..

[40] Newberry S.J., Fitzgerald J.D., Maglione M.A. (2015). Systematic review for effectiveness of hyaluronic acid in the treatment of severe degenerative joint disease (DJD) of the knee.

[41] Wobig M., Dickhut A., Maier R., Vetter G. (1998). Viscosupplementation with Hylan G-F 20: A 26-week controlled trial of efficacy and safety in the osteoarthritic knee.. Clin. Ther..

[42] Mackowiak J., Jones J.T., Dasa V. (2020). A comparison of 4-year total medical care costs, adverse outcomes, and opioid/prescription analgesic use for 3 knee osteoarthritis pain treatments: Intra-articular hyaluronic acid, intra-articular corticosteroids, and knee arthroplasty.. Semin. Arthritis Rheum..

[43] Waddell D., Rein A., Panarites C., Coleman P.M., Weiss C. (2001). Cost implications of introducing an alternative treatment for patients with osteoarthritis of the knee in a managed care setting.. Am. J. Manag. Care.

[44] Waddell D.D., Joseph B. (2016). Delayed total knee replacement with hylan G-F 20.. J. Knee Surg..

[45] Ong K.L., Runa M., Lau E., Altman R. (2019). Is intra-articular injection of synvisc associated with a delay to knee arthroplasty in patients with knee osteoarthritis?. Cartilage.

[46] Dasa V., Lim S., Heeckt P. (2018). Real-world evidence for safety and effectiveness of repeated courses of hyaluronic acid injections on the time to knee replacement surgery.. Am. J. Orthop..

[47] Altman R., Lim S., Steen R.G., Dasa V. (2015). Hyaluronic acid injections are associated with delay of total knee replacement surgery in patients with knee osteoarthritis: Evidence from a large U.S. Health claims database.. PLoS One.

[48] Concoff A., Niazi F., Farrokhyar F., Alyass A., Rosen J., Nicholls M. (2021). Delay to TKA and costs associated with knee osteoarthritis care using intra-articular hyaluronic acid: Analysis of an administrative database.. Clin. Med. Insights Arthritis Musculoskelet. Disord..

[49] Mordin M., Parrish W., Masaquel C., Bisson B., Copley-Merriman C. (2021). Intra-articular hyaluronic acid for osteoarthritis of the knee in the United States: A systematic review of economic evaluations.. Clin. Med. Insights Arthritis Musculoskelet. Disord..

[50] Waddell D.D., Bricker D.C. (2007). Total knee replacement delayed with Hylan G-F 20 use in patients with grade IV osteoarthritis.. J. Manag. Care Pharm..

[51] Delbarre A., Amor B., Bardoulat I., Tetafort A., Pelletier-Fleury N. (2017). Do intra-articular hyaluronic acid injections delay total knee replacement in patients with osteoarthritis – A Cox model analysis.. PLoS One.

[52] Altman R., Fredericson M., Bhattacharyya S., Bisson B., Abbott T., Yadalam S., Kim M. (2015). Association between hyaluronic acid injections and time-to-total knee replacement surgery.. J. Knee Surg..

[53] Altman R.D., Manjoo A., Fierlinger A., Niazi F., Nicholls M. (2015). The mechanism of action for hyaluronic acid treatment in the osteoarthritic knee: A systematic review.. BMC Musculoskelet. Disord..

[54] Hashizume M., Mihara M. (2010). High molecular weight hyaluronic acid inhibits IL-6-induced MMP production from human chondrocytes by up-regulating the ERK inhibitor, MKP-1.. Biochem. Biophys. Res. Commun..

[55] Julovi S.M., Ito H., Nishitani K., Jackson C.J., Nakamura T. (2011). Hyaluronan inhibits matrix metalloproteinase-13 in human arthritic chondrocytes *via* CD44 and P38.. J. Orthop. Res..

[56] Waddell D.D., Kolomytkin O.V., Dunn S., Marino A.A. (2007). Hyaluronan suppresses IL-1beta-induced metalloproteinase activity from synovial tissue.. Clin. Orthop. Relat. Res..

[57] Henrotin Y., Chevalier X., Deberg M., Balblanc J.C., Richette P., Mulleman D., Maillet B., Rannou F., Piroth C., Mathieu P., Conrozier T. (2013). Early decrease of serum biomarkers of type II collagen degradation (Coll2-1) and joint inflammation (Coll2-1 NO _2_ ) by hyaluronic acid intra-articular injections in patients with knee osteoarthritis: A research study part of the Biovisco study.. J. Orthop. Res..

[58] Hummer C.D., Angst F., Ngai W., Whittington C., Yoon S.S., Duarte L., Manitt C., Schemitsch E. (2020). High molecular weight Intraarticular hyaluronic acid for the treatment of knee osteoarthritis: A network meta-analysis.. BMC Musculoskelet. Disord..

[59] Donovan R.L., Edwards T.A., Judge A., Blom A.W., Kunutsor S.K., Whitehouse M.R. (2022). Effects of recurrent intra-articular corticosteroid injections for osteoarthritis at 3 months and beyond: A systematic review and meta-analysis in comparison to other injectables.. Osteoarthritis Cartilage.

[60] Bannuru R.R., Natov N.S., Obadan 
I.E.
, Price L.L., Schmid C.H., McAlindon T.E. (2009). Therapeutic trajectory of hyaluronic acid *versus* corticosteroids in the treatment of knee osteoarthritis: A systematic review and meta-analysis.. Arthritis Care Res..

[61] Kompel A.J., Roemer F.W., Murakami A.M., Diaz L.E., Crema M.D., Guermazi A. (2019). Intra-articular corticosteroid injections in the hip and knee: Perhaps not as safe as we thought?. Radiology.

[62] McAlindon T.E., LaValley M.P., Harvey W.F., Price L.L., Driban J.B., Zhang M., Ward R.J. (2017). Effect of intra-articular triamcinolone *vs* saline on knee cartilage volume and pain in patients with knee osteoarthritis.. JAMA.

[63] Habib G., Khazin F., Chernin M. (2014). Continuous blood glucose monitoring in a patient with type 2 diabetes who underwent intraarticular betamethasone injection for knee osteoarthritis.. Arthritis Rheumatol..

[64] Habib G.S., Bashir M., Jabbour A. (2008). Increased blood glucose levels following intra-articular injection of methylprednisolone acetate in patients with controlled diabetes and symptomatic osteoarthritis of the knee.. Ann. Rheum. Dis..

[65] Habib G.S., Miari W. (2011). The effect of intra-articular triamcinolone preparations on blood glucose levels in diabetic patients: A controlled study.. J. Clin. Rheumatol..

[66] Navarro-Sarabia F., Coronel P., Collantes E., Navarro F.J., de la Serna A.R., Naranjo A., Gimeno M., Herrero-Beaumont G. (2011). A 40-month multicentre, randomised placebo-controlled study to assess the efficacy and carry-over effect of repeated intra-articular injections of hyaluronic acid in knee osteoarthritis: The AMELIA project.. Ann. Rheum. Dis..

[67] Johnston J., Brown K., Muir J., Sloniewsky M.J. (2021). Long-term outcomes of single *versus* multiple courses of viscosupplementation for osteoarthritic knee pain: Real-world, multi-practice experience over a six-year period.. J. Pain Res..

[68] Bhadra A.K., Altman R., Dasa V., Myrick K., Rosen J., Vad V., Vitanzo P., Bruno M., Kleiner H., Just C. (2017). Appropriate use criteria for hyaluronic acid in the treatment of knee osteoarthritis in the United States.. Cartilage.

[69] Spitzer A.I., Bert J.M. (2018). Letter to the Editor: Compliance with the aaos guidelines for treatment of osteoarthritis of the knee: A survey of the American association of hip and knee surgeons.. J Am Acad Orthop Surg.

[70] Carlson V.R., Ong A.C., Orozco F.R., Hernandez V.H., Lutz R.W., Post Z.D. (2018). Compliance with the AAOS guidelines for treatment of osteoarthritis of the knee: A survey of the american association of hip and knee surgeons.. J. Am. Acad. Orthop. Surg..

[71] Conrozier T., Monfort J., Chevalier X., Raman R., Richette P., Diraçoglù D., Bard H., Baron D., Jerosch J., Migliore A., Henrotin Y. (2020). EUROVISCO recommendations for optimizing the clinical results of viscosupplementation in osteoarthritis.. Cartilage.

